# Interactions between rnacrophage cytokines and eicosanoids in expression of antitumour activity

**DOI:** 10.1155/S0962935192000449

**Published:** 1992

**Authors:** Shlomo Ben-Efraim

**Affiliations:** 1 Department of Pharmacology Faculty of Medicine Erasmus University Rotterdam 3000 DR The Netherlands; 2 Department of Human Microbiology Sackler School of Medicine TeI-Aviv University TeI-Aviv 69978 Israel

## Abstract

Cytokines and eicosanoid products of macrophages play an essential role in expression of antitumour activity of macrophages either in a cell-to-cell contact system between the effector and the target cell or as cell-free soluble products. In this review the relationship between three main monokines, namely TNF-α, IL-1 and IL-6 and the interrelationship between these monokines and eicosanoids (PGE_2_, PGI_2_, LTB_4_, LTC_4_) in their production and in expression of antitumour activity is discussed. Emphasis is given to the effect of tumour burden on production of the monokines and of the eicosanoids and on the production of these compounds by the tumour cells. Finally, the therapeutic implications drawn from animal studies and clinical trials is discussed.


Invited Review

Mediators of Inflammation, 1, 295–308 (1992)

### Interactions between macrophage cytokines and eicosanoids in expression of antitumour activity

Shlomo Ben-Efraim

Department of Pharmacology, Faculty of Medicine, Erasmus University, 3000 DR Rotterdam, The Netherlands
Permanent Address: Department of Human Microbiology, Sackler School of Medicine, Tel-Aviv University, Tel-Aviv 69978, Israel

**CYTOKINES and eicosanoid products of macrophages play an essential role in expression of antitumour activity of macrophages either in a cell-to-cell contact system between the effector and the target cell or as cell-free soluble products. In this review the relationship between three main monokines, namely TNF-α, IL-1 and IL-6 and the interrelationship between these monokines and eicosanoids ($PGE_2$, $PGI_2$, $LTB_4$, $LTC_4$) in their production and in expression of antitumour activity is discussed. Emphasis is given to the effect of tumour burden on production of the monokines and of the eicosanoids and on the production of these compounds by the tumour cells. Finally, the therapeutic implications drawn from animal studies and clinical trials is discussed.**

**Key words:** Antitumour activity, Cancer immunotherapy, Cytokines, Eicosanoids, IL-1, IL-6, Leukotrienes, $LTB_4$, $LTC_4$, Macrophages, Monokines, $PGE_2$, Prostaglandins, TNF-α

### Introduction

Since the pioneering work of Metchnikoff,[1] it became more and more evident that macrophage cells play an essential role in a wide array of biological activities. This includes among others the events of nonspecific resistance against invasion of foreign cells (including tumour cells), their function as a crucial mediator in the development of immune response and participation in the process of inflammation (for review see Ref. 2).

The various functions of macrophages are exerted either in a cell-to-cell contact set-up with the target cells or by various biologically and pharmacologically active factors released by these cells. Among the most salient factors released by macrophages are various monokines and products of arachidonic acid (prostaglandins and leukotrienes).

An extensive amount of work has been done on the pharmacological and biological effects of macrophage cytokines and eicosanoids. The present review is by no means intended to provide a full coverage of all the activities of macrophage cytokines and eicosanoids. The aim is to discuss only the topic of interrelation between certain macrophage cytokines and eicosanoids in the context of their expression of antitumour activity. The main emphasis will be on the role of macrophage cytokines tumour necrosis factor-α (TNF-α) interleukin-1 (IL-1) and interleukin-6 (IL-6) and on the role of prostaglandin $E_2$ ($PGE_2$), leukotriene $B_4$ ($LTB_4$) and leukotriene $C_4$ ($LTC_4$) and their interrelation in the production and expression of antitumour activity. Another aspect to be discussed is the effect of tumour burden on their production by macrophages, production of these compounds by tumour cells and their therapeutic effectiveness in experimental tumour models and in cancer patients.

The first indications on induced release of certain antitumour factors by bacteria-free filtrates came for the early work of Coley (for review see Ref. 3). Later on, Carswell *et al.*[4] reported the occurrence of an antitumour cytotoxic factor in the serum of mice which had undergone treatment with bacterial lipopolysaccharide (LPS), coined the term tumour necrosis factor (TNF) and suggested that TNF is produced by activated macrophages in response to LPS. The occurrence of a lymphocyte activated factor produced by macrophages was first reported in 1972[5,6] and was named lymphocyte activating factor (LAF). In 1979, the nomenclature of various cytokines was revised[7] and the former LAF was given the name of IL-1 which is still used today. IL-6 was initially described as a factor derived from fibroblasts with antiviral activity.[8] The term IL-6 was first suggested by Poupart and colleagues[9] and its production by human monocytes was reported.[10]

### Interactions in the production of macrophage cytokines and eicosanoids

*Interactions in production between TNF-α, IL-1 and IL-6:* It has been reported that production from blood mononuclear cells and from peritoneal macrophages of TNF-α, IL-1 and IL-6, can be induced by the same stimulatory agents. This applies to LPS,[11–13] phytohaemagglutinin (PHA)[13]

© 1992 Rapid Communications of Oxford Ltd and *Staphylococcus epidermis*.[13] It should be noted also that the same monokine can be induced by different mechanisms. Thus, it was reported that *Mycoplasma capricolum* membranes induced TNF-α by a mechanism different from induction by LPS.[14]

In spite of the similarities in stimulation of the production of the monokines, some differences were reported in the context of their production. These differences indicate that mechanisms of their production may be different. Thus, human monocytes stimulated with pneumococcal cell surface components produced IL-1 but not TNF-α.[15] Both protein kinase C (PKc) and calmodulin (CaM) kinase dependent pathways were found to be involved in the induction of IL-1 mRNA by LPS, whereas TNF-α expression seemed to be PKc dependent but not CaM kinase dependent.[16] Other authors reported that TNF-α and IL-1 production and secretion by mononuclear phagocytes can be modulated differentially.[17] Differences between the kinetics of production of IL-1α, IL-1β and TNF-α by murine peritoneal macrophages during the peritoneal exudative response, were also described in relation to the optimal culture conditions and sequence of appearance.[18] The production of TNF-α and IL-1 in alveolar human macrophages was found to be regulated differentially by LPS.[19] A different pattern of regulation was also observed in the case of human macrophages: during the initial phase of maturation of human blood monocytes (up to 7 days in culture), IL-1β and IL-6 were down-regulated whereas TNF-α levels markedly increased.[20] A synergistic effect of interferon-τ (IFN-τ) and LPS was observed in relation to the release of IL-1β, IL-6 and TNF-α from human macrophages.[20] However, the LPS-induced levels of these cytokines differed during prolonged cultivation of macrophages (up to 28 days).[20] Differences in levels of production of IL-1α, IL-1β and TNF-α versus lower levels of IL-6 were also reported following stimulation by various agents of human blood mononuclear cells.[13]

The relationship in production between TNF-α, IL-1β and IL-6 is expressed by the findings that each one of these monokines can affect the production of the other monokines. Thus TNF-α was found to induce release of IL-1 *in vitro*[21,22] and *in vivo*.[23] TNF-α and IL-1 induced IL-6 production *in vivo*.[24] Stimulation of human monocytes by IL-1 caused a rapid down-regulation of IL-6 mRNA levels and concomitant enhancement of IL-6 mRNA expression.[24] IL-6 itself was found to suppress the IL-6-R at high concentrations.[25] IL-6 suppressed IL-1β and TNF-α production induced by LPS or PHA in human blood mononuclear cells.[13] Inhibition of LPS-induced TNF production by IL-6 in cultured human monocytes was also reported by other authors.[26] By working with bone marrow derived mouse macrophages it was found that LPS induces secretion of both TNF-α, IL-1 and IL-6.[27] IL-1 was able to stimulate IL-6 synthesis in human blood monocytes but not in monocyte derived macrophages whereas TNF-α had no effect on IL-6 synthesis in monocytes or macrophages.[28] Human alveolar macrophages and blood monocytes produced large amounts of IL-6 in response to LPS and monocytes produced lesser amounts of IL-6 in response to rIL-1.[29] Monocytes aged *in vitro* produced little detectable IL-6 in response to LPS or rIL-1, which might suggest that release of IL-6 under stimulus is correlated to the degree of maturity of macrophage cells.[29] TNF-α or rIL-6 itself did not modulate IL-6 production in human peripheral blood mononuclear cells.[30] No evidence was found that TNF-α acts to amplify the production of IL-6 or IL-1 by murine macrophage cell lines.[31] It was also claimed that synthesis and secretion of IL-1 either by human monocytes[32] or by mouse bone marrow derived macrophages[33] are two different biological events.

Similarly, LPS-induced production of TNF-α[34] and IL-1[35] was also reported in human peritoneal macrophages collected from patients on continuous ambulatory peritoneal dialysis (CAPD) during an episode of infectious peritonitis.

The conclusion from the above-mentioned data is that TNF-α, IL-1 and IL-6 can mutually affect their production. The production and release of these cytokines are also affected by other agents (including other cytokines) but these findings are beyond the scope of the present review. A schematic representation of the interrelationship in production between the three cytokines is given in Fig. 1.

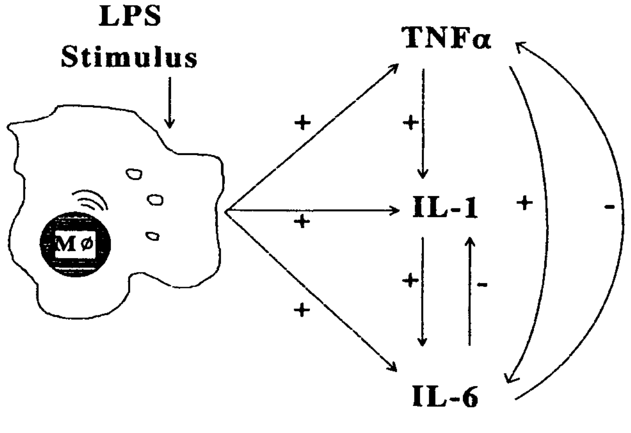

FIG. 1. Schematic representation of interrelationship in production by macrophages between TNFα, IL-1 and IL-6. +: enhancement; −: inhibition.

*Interactions in production between cytokines and eicosanoids:* A series of findings indicated that production of TNF-α by macrophages can be regulated by both endogenous and exogenous $PGE_2$. Thus, in response to LPS, murine peritoneal macrophages release concomitantly increased amounts of TNF-α and $PGE_2$.[36,37] However, addition of exogenous $PGE_2$ strongly suppressed the release of TNF-α by macrophages.[36,37] $PGE_2$ down-regulated the expression of TNF-α gene in human blood monocytes.[38] In another work it was reported that low amounts of $PGE_2$ enhance release of TNF-α from macrophages whereas high doses of $PGE_2$ suppress its release.[39] Some authors reported recently that the gene expression of TNF-α was enhanced by low doses of $PGE_2$ and by cGMP, and suggested that cGMP may represent one of the positive signals for TNF-α synthesis.[40]

Suppression of LPS-induced TNF-α production by $PGE_2$ was also reported by other authors.[41,42] The inhibitory effect of $PGE_2$ on TNF-α production was correlated with induced augmentation of cAMP in macrophage cells.[36,39] In contrast to $PGE_2$, leukotrienes induce increases in TNF-α release from macrophages. Thus, human monocytes exposed to graded concentrations of $LTB_4$ release high amounts of TNF-α.[43] The enhancing effect of leukotrienes on TNF-α may be related to increases in cGMP levels by leukotrienes[44] and is also supported by findings that lipoxygenase inhibitors suppress formation of TNF-α *in vitro* and *in vivo*.[45] Treatment of macrophages with LPS enhanced the increase of a lipoxygenase product which counteracted the suppression of TNF-α synthesis by a lipoxygenase inhibitor when added to macrophages exogenously.[46] Recent data suggest that endogenous prostaglandins and leukotrienes do not play a role in the regulation of TNF-α production.[47] In addition indomethacin (IND) (a cyclooxygenase inhibitor), exogenous arachidonate and MK-886 (a novel inhibitor of 5-lipoxygenase product formation) do not affect TNF-α production.[47] The interrelationship between TNF-α and $PGE_2$ production is also supported by the finding that TNF-α stimulates $PGE_2$ production in murine resident peritoneal macrophages.[22] Other data indicate that enhancement of TNF-α activity may be independent of $PGE_2$ production. Thus IFN-γ in combination with LPS enhanced TNF-α production but addition of IFN-γ to LPS had no effect on $PGE_2$ levels produced in human monocytes.[48]

With regard to IL-1, it was found that the same stimulator, namely PHA, induced production of both IL-1 and prostaglandin E in human monocyte monolayers. Products of the cyclooxygenase pathway of arachidonate metabolism seem not to be involved in the mechanism by which IL-1 stimulates thymocyte proliferation, whereas products of the lipoxygenase pathway may mediate the thymocyte proliferative response induced by IL-1. Similarly, with TNF-α, it seems that an arachidonate lipoxygenase product is important in the sequence of events leading to the production of IL-1. Additionally lipoxygenase inhibitors affected production of IL-1 in human peripheral blood monocytes.[49] An enhancing role of leukotrienes in production of IL-1 was also advocated by other authors.[50,51] Recently, it was reported that addition of exogenous $LTB_4$ to monocytes stimulates IL-1β transcription and mRNA accumulation.[52] A self-regulatory mechanism of IL-1 production was suggested by data showing that exogenous IL-1 induces increases in the levels of $PGE_2$ in murine macrophage cultures, whereas exogenous $PGE_2$ or prostacyclin ($PGI_2$) (measured as its stable metabolite 6-keto prostaglandin $F_{1\alpha}$) suppressed macrophage IL-1 production.[53] The inhibitory effect of $PGE_2$ was correlated to induce increases in cAMP levels.[54] IL-1 was found, on the other hand, to stimulate 5-lipoxygenase activity and in this way induces increases in $PGE_2$ synthesis.[55] In contrast to these data, it was also reported[56] that $PGE_2$ had no effect on IL-1 synthesis in murine resident peritoneal macrophages but rather had a direct inhibitory effect on thymocyte proliferation.[57]

In spite of the similarities in the stimulating conditions for production of TNF-α and IL-1 some differences were also reported. Thus, it was claimed that $PGE_2$ suppresses expression of cell-associated TNF-α in murine peritoneal macrophages but had no effect on cell-associated IL-1 activity.[58] $PGE_2$ suppressed accumulation of TNF mRNA but not of IL-1α and IL-1β mRNA accumulation.[58] The conclusion from these experiments was that synthesis of TNF appears to be regulated at the level of transcription whereas synthesis of IL-1α and IL-1β is regulated post-transcriptionally.[58] Recently, it was shown that $PGE_2$ inhibits release of TNF-α but not of IL-1β from human peritoneal macrophages.[42] Interrelation between production of prostaglandins and of IL-1β in human peritoneal macrophages was recently reexamined.[59] It was found that $PGI_2$ (measured as its stable metabolite 6-keto-$PGF_{1\alpha}$) declined sharply during episodes of peritonitis both in the presence or absence of LPS in the culture medium of human peritoneal macrophages.[59] On the other hand, $PGE_2$ was released in the same amounts in cultures of macrophages collected during peritonitis and during an infection-free period.[59] These results suggest that $PGI_2$ and $PGE_2$ may play a different role in the regulation of IL-1β production by human macrophages.[59]

The production of IL-6 concomitantly to production of $PGE_2$ in LPS-stimulated rat Kupffer cells was examined.[60] IL-6 production increased in

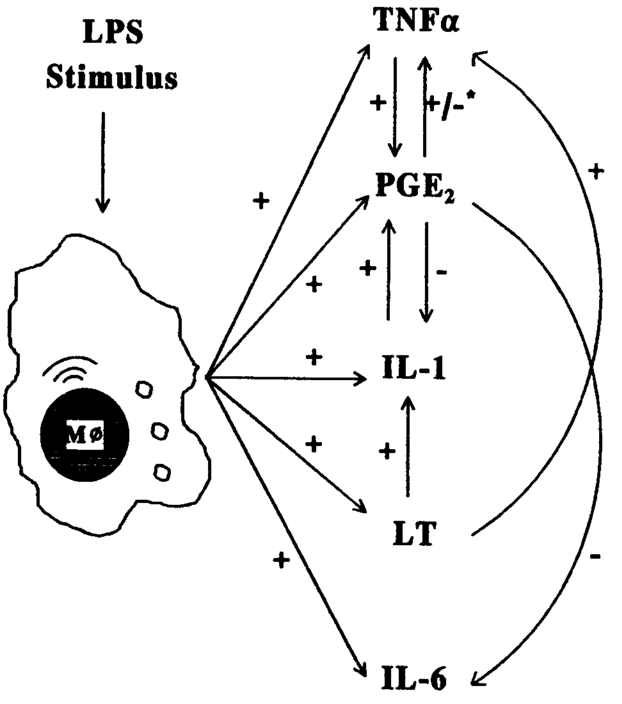

FIG. 2. Schematic representation of interrelationship in production by macrophages between cytokines (TNFα, IL-1 and IL-6) and eicosanoids ($PGE_2$; LT: leukotrienes); +: enhancement; −: inhibition; *+ at low doses, − at high doses.

parallel with $PGE_2$ before decreasing as $PGE_2$ continued to rise.[60] Blocking of $PGE_2$ production by IND increased IL-6 levels significantly thus showing that $PGE_2$ produced by Kupffer cells down-regulates IL-6 secretion.[60] However, cyclooxygenase inhibitors inhibited production of IL-6 by human peripheral blood mononuclear cells,[61] but no direct relationship between inhibition of IL-6 and production of $PGE_2$ was found.[61] Leukotrienes stimulate production of IL-6 in cultures of human monocytes.[62,63] $LTB_4$ stimulates production of IL-6 and induces accumulation of IL-6 mRNA.[62,63] Finally, regarding the interrelation between the production of IL-1 and $PGE_2$, it was reported that IL-1 and $PGE_2$ are produced by separate subsets of human monocytes.[64] The interrelationship between the production of cytokines and eicosanoids is given in Fig. 2.

### Effect of tumour burden on production of macrophage cytokines and eicosanoids

The functions of tumour associated macrophages (TAM) have been investigated extensively (for reviews see Refs. 65, 66). It was suggested that *in situ* macrophages may affect the biology of neoplastic tissues in various ways besides tumour killing, by producing growth factors, by interaction with haemostatic mechanisms, by release of mutagenic reactive oxygen intermediates and neutral proteinases, and by their capacity to induce angiogenesis.[65]

The effect of tumour burden on production of macrophage cytokines and eicosanoids was examined in a series of experimental systems and in cases of human cancer.

*Experimental studies:* It was reported that during tumour growth in rats (subcutaneous implantation), cyclooxygenase or thromboxane synthase is inhibited, whereas C5 and C12-lipoxygenases of the alveolar macrophages are activated.[67,68] A transient increase of 12-HETE and $LTC_4$ production in murine peritoneal macrophages was also reported in mice implanted subcutaneously with B16 melanoma cells.[69] Tumour–host derived macrophages were found to suppress a series of events including activation of T cells, natural killing (NK) cells and lymphocyte activated killer (LAK) cells, and generation of tumoricidal activity in normal syngeneic murine splenic macrophages cultures in the presence of LPS.[70] The suppressive effects were correlated with an increase in $PGE_2$ secretion by tumour-bearing host macrophages.[70]

The role of prostaglandin secretion by macrophages from mice bearing syngeneic tumours was also supported by other authors. Thus, splenic and peritoneal macrophages collected from mice bearing different syngeneic tumours secreted large amounts of $PGE_2$ as a result of their interaction with the tumours.[71] These macrophages were immunosuppressive and their suppressive activity was significantly reduced by IND thus proving that the suppressive effect was due to increased release of prostaglandin.[71] In another study, it was shown that inhibition of spleen cell cytotoxic capacity toward murine Lewis carcinoma was due to an increase in $PGE_2$ levels.[72] The inhibitory effect was prevented by pretreatment of tumour-bearing mice with IND.[72]

The effect of tumour burden on the ability of macrophages to secrete cytokines was also examined. Continuous alveolar macrophage (AM) and tumour infiltrated (TIM) cell lines were generated from C57BL/6J mice and tested for their potential to secrete IL-1, TNF-α or IL-1 following exposure to rMulFN-γ and LPS.[73] Neither cell line secreted substantial amounts of IL-1 or TNF-α but secreted large amounts of IL-6.[73] Peritoneal macrophages from sarcoma-bearing mice produced progressively less IL-1 as tumour burden increased.[74] Administration of LPS to tumour-bearing mice early after tumour transplantation, resulted in reduced tumour growth and prevented the down-regulation of *in vitro* IL-1 production by peritoneal macrophages.[74] Thus, it seems that a specific defect in IL-1 production was associated with increasing tumour burden.[74] In another study it was concluded that murine tumour-infiltrating macrophages isolated from the lungs of mice bearing lung B16F10 metastases responded as normal alveolar macrophages to biological response modifiers in relation to induction of tumoricidal activity and to secretion of IL-1 and TNF-α.[75] However, tumour associated macrophages (TAM) isolated from five cases of murine sarcomas showed a limited capacity to produce and release IL-1 as compared to peritoneal macrophages upon stimulation with LPS.[76]

The role of TNF-α release by TAM in specific defence against the inoculated tumour was questioned by various authors. Thus TAM from mice bearing EMT6 tumours exhibited high anti-WEHI-164 activity[77,78] due to release of TNF-α[78] but was not effective against the EMT6 tumour.[77,78] Similarly, TAM in a murine fibrosarcoma model produced TNF-α but this production did not affect the tumour growth.[79]

*Human studies:* Indirect indication of the role of prostaglandin production by macrophages from cancer patients was provided by data showing that monocyte-derived macrophages isolated from blood of cancer patients can be rendered cytotoxic by treatment with IND.[80,81] Apparently not all macrophages collected from cancer patients could be rendered cytotoxic against allogeneic or autologous tumour target cells, presumably because they were nonresponsive and/or because of the presence of a plasma inhibitory factor.[81] In another work, it was reported that peripheral and bone marrow enriched fraction of monocytes produce high levels of $PGE_2$.[82] However, the increased release of $PGE_2$ was not correlated to clinical or pathological findings.[82]

The production of IL-1 and TNF-α by tumour-associated mononuclear leukocytes (TAML) and peripheral blood mononuclear leukocytes (PBML) in cancer patients was determined. Stimulation by LPS induced production of similar levels of TNF-α in TAML and PBML but production of IL-1 was markedly suppressed in LPS-stimulated tumour-associated mononuclear leukocytes (TAML).[83] In another study the release of IL-1 and IL-6 by TAM from ascites and solid tumours of human ovarian carcinoma was investigated. It was found that TAM release spontaneously or upon LPS stimulation high amounts of IL-6 whereas they were poor producers of IL-1.[84] LPS-induced production of TNF-α by peripheral blood macrophages was impaired in cases of breast cancer.[85] On the other hand, secretion of TNF-α by monocytes from patients with malignant brain tumours was significantly greater by comparison with monocytes from normal individuals.[86] Increase in secretion of TNF-α and of IL-1 was also reported by alveolar macrophages from patients with lung cancer as compared with secretion by peripheral blood monocytes from the same patients or by alveolar macrophages from patients with nonmalignant disorders.[87] However, alveolar macrophages from lung cancer patients were found to be impaired in their ability to develop antitumour cytotoxic activity compared with either the peripheral blood monocytes from the same patients or alveolar macrophages from patients with nonmalignant lung disorders.[87] An increase in the level of TNF-α was also found in tumour-infiltrating macrophages in human colorectal adenocarcinoma.[88] The increase in TNF-α levels correlated with an increase in the size of the primary tumour.[88]

Some work was dedicated to determining the interrelationship between the production of TNF-α and $PGE_2$ by monocytes from cancer patients. LPS-incubated monocytes from cancer patients with malignancies of the digestive tract, produced high levels of TNF-α and $PGE_2$ when cultured in medium with foetal bovine serum.[89] Addition of cancer-patient plasma to the medium suppressed markedly TNF production but induced a prominent enhancement of $PGE_2$ production.[89] Plasma of cancer patients did not exhibit TNF-α activity but such plasma contained increased levels of $PGE_2$.[89] However, although low amounts of exogenous $PGE_2$ suppressed TNF-α production by normal monocytes, addition of 10% plasma-containing $PGE_2$ did not induce suppression of TNF-α production, thus indicating that some unidentified factor(s) in the plasma of cancer patients modulates the TNF-α and $PGE_2$ production in these patients.[89] Correlation between production of TNF-α and $PGE_2$ by peripheral blood monocytes was also studied in patients with bladder cancer. It was found that these patients had either higher TNF-α production or higher $PGE_2$ production.[90] A shift in macrophage population was due to tumour growth in BALB/c mice: immunosuppression in the tumour-bearing host was caused at least in part to the inability of $Mac\text{-}1^+$ and/or $Mac\text{-}3^+$ to control production of $PGE_2$ by $Mac\text{-}2^+$ macrophages.[91] Certain tumour-cell membrane constituents were found to activate human monocytes for TNF-α synthesis.[92]

In view of the wide variation in the results on release of macrophage cytokines and prostaglandin by monocyte-macrophages and tumour-associated macrophages from cancer patients, it still seems difficult to draw final conclusions on the role of this release in clinical settings of neoplasia. It seems that increased release of $PGE_2$ is usually found in TAM and may be correlated to an increase in the severity of the disease. Thus, high prostaglandin production in tissues surrounding human breast tumours is correlated with high metastatic potential for neoplastic cells.[93] Successful therapy with IND and with a combination of IND and IL-2 was explained by abrogation of prostaglandin-mediated suppression of NK activity and IL-2 production.[94-97]

### Production of eicosanoids and cytokines by tumour cells

In view of the role played by eicosanoids and cytokines in expression of antitumour activity it seems likely that intrinsic production of these compounds by tumour cells may affect the resistance to tumour development in tumour-bearing animals and in human neoplasia.

First reports indicated that $BP8/P_1$ murine ascitic tumour and, to a less extent a subcutaneously implanted S180 rat tumour, produced $PGE_2$.[98] However, the role of $PGE_2$ production in the development of the tumour remains uncertain because induction of a decrease in $PGE_2$ levels by IND did not affect appreciably the tumour growth.[98] In more recent work it was shown that certain murine tumours produce prostaglandins and that their response to IND therapy was directly related to their ability to produce prostaglandin.[99] Production of $PGE_2$ by EL4 leukaemia cells from C57BL/6 mice was also correlated to the extent of migration and dissemination of the tumour.[100] Prostaglandin biosynthesis was also found to occur in established cell lines derived from human lung, colorectal adenocarcinoma, and ovarian adenocarcinoma.[101] A difference in the amount of $PGE_2$ released was found between cancer cells metastasizing into rat liver or rat kidney.[102] It was assumed that this difference may be related to the mechanism of cancer metastases or to selection of the organ in which metastases occur.[102]

Tumour cells were reported to also produce cytokines. Thus, tumours from cachectic mice produced both TNF-α and IL-1α *in vivo* as documented by the presence of TNF-α and IL-1α mRNA and immune-reactive protein for IL-1α.[103] The tumour cells also produced TNF-α and IL-1α in long-term cultures but not IL-6.[103] Secretion of TNF-α by human leukaemic cells was also reported.[104] A myeloma cell line established from the pleural effusion of a myeloma patient secreted both TNF-α and IL-6 and these cytokines induced proliferation of the cell line.[105] In IL-6 production a dual effect was described: the murine MH134 tumour cells produced high amounts of IL-6 whereas the murine CSA1M tumour produced only marginal levels of IL-6.[106] However, both tumours induced production of IL-6 by T cells in the tumour-bearing host.[106] Leukaemic cells from patients with acute myeloid leukaemia produced both IL-6 and IL-1.[107] Prostatic carcinoma cell lines expressed the IL-6 receptor and secreted IL-6.[108] Squamous cell carcinoma cell lines produced both IL-1 and IL-6.[109]

An interesting situation was described with the murine P815 mastocytoma: this line produces TNF[110] but, at the same time, this was one of the first tumour cell lines which was found to be sensitive to exogenous TNF.[3,4]

It seems likely that production of prostaglandins and macrophage cytokines as well as induction of their production by macrophages in tumour-bearing hosts plays a role in the development of the tumour *in vivo*. However, the direct relationship between neoplasia and the ability of tumour cells to produce and/or induce production of macrophage eicosanoids and cytokines is not clear yet.

### Interactions between macrophage cytokines and eicosanoids in expression of antitumour activity

*Interactions between TNF-α, IL-1 and IL-6:* TNF-α alone is cytostatic or cytotoxic for a wide range of murine and human tumour-cell lines.[111,112] On the other hand, TNF had no effect on a wide variety of murine and human tumour-cell lines and enhanced the growth of various normal cell lines.[111,112] Moreover, a heterogeneous cytotoxic response of TNF-α was described for various cell lines isolated from the same single neoplasm of human colorectal carcinoma[113] or renal cell carcinoma.[114] Membrane-associated TNF was shown to be the lytic principle of activated macrophages cytotoxic for TNF susceptible tumour cells.[115] The complexity of TNF antitumour activity is also shown by findings that TNF-α mediates the enhanced cytotoxicity induced in monocytes by IFN, IL-1 and by TNF itself.[116] Treatment of human monocytes with TNF-α increased their cytostatic ability in a dose-dependent manner against P815 murine mastocytomas.[117] IL-1 was also reported to be cytocidal for several tumour-cell lines.[118] However, IL-1 can act also as an autocrine growth factor for acute myeloid leukaemia cells.[119]

The fact that TNF-α and IL-1 are both produced by activated macrophages, and that TNF-α itself is an inducer of IL-1 production, prompted investigations devised to determine possible synergistic and additive antitumour effects of combinations of the two cytokines. Combination of TNF-α and IL-1 synergistically[120] or in additive manner[121] inhibited the growth of human A-375 melanoma cells. In another study, it was claimed that enhancement of antitumour human monocyte activity by combined TNF-α and IL-1β was less than additive.[122] We found that a combination of TNF-α and IL-1 had an additive effect on antitumour cytostasis against WEHI-3B murine tumour cells.[123] It should be noted that TNF-α and IL-1 represent only a fraction of a wider spectrum of macrophage cytokines involved in expression of antitumour activity of macrophages.[124,125]

Antitumour activity of IL-6 against human breast carcinoma and leukaemia/lymphoma cell lines[126] and *in vivo* against four murine metastatic tumours[127] has been reported. On the other hand, autocrine generation and requirement as a growth factor for human multiple myelomas[128] and for human renal cell carcinomas[129] has also been described.

IL-6 production is induced by LPS which also stimulates production of TNF-α and IL-1, IL-1 induced synthesis of IL-6 in human blood monocytes[28,29] and TNF-α induced IL-6 in sera of cancer patients and tumour-bearing mice.[130] Moreover, it was found that IL-6 is involved in IL-1 induced activities as pyrogenicity and stimulation of thymocyte proliferation.[131] However, there are apparently few data on synergistic, additive or antagonistic effects of combination of IL-6 with either TNF-α or IL-1 on tumour cells. It was claimed that systemic administration of low doses of IL-6 in combination with sub-therapeutic doses of TNF to mice bearing a weakly immunogenic syngeneic tumour resulted in marked regression and some cure.[127]

*Interactions between cytokines and eicosanoids:* Modulation of production of various arachidonic acid derivatives is by itself related to induction of antitumour activity in macrophages. Thus, *in vitro* treatment of murine peritoneal macrophages with IND, a cyclooxygenase inhibitor, induced antitumour cytostatic activity against a murine plasmacytoma and this effect was increased when $LTB_4$ was added to the cultures.[132,133] The IND stimulation of macrophage cytostasis against the murine plasmacytoma was enhanced by endogenous metabolites of lipoxygenase and counteracted by $PGE_2$.[134] Induction of macrophage cytostasis towards P815 mastocytoma by calcium ionophore was reversed by specific inhibition of lipoxygenase.[135] In other work it was shown that $LTC_4$ is an essential 5-lipoxygenase intermediate in A23187-induced antitumour cytostatic activity[136] and that addition of L-serine to cultures stimulated by calcium ionophore increased both the accumulation of $LTC_4$ in murine macrophages as well as their antitumour activity.[137] LPS-induction of macrophage tumour killing was counteracted by $PGE_2$ but not by $PGI_1$.[138]

It should be noted that there are contradictory reports concerning the effect of prostaglandin production by macrophages tumour cells, and the direct effect of prostaglandins on tumour growth. Thus, it was reported that PGE could inhibit DNA synthesis and tumour cell replication *in vitro* and tumour growth *in vivo*.[139,140] PGA also inhibited tumour growth *in vitro* and *in vivo*.[141,142] However, in spite of the fact that induction of antitumour activity in macrophages by LPS was associated with an increase in $PGE_2$ and thromboxane production, these compounds did not seem essential for the expression of antitumour activity, as induction of antitumour activity took place and was even enhanced in the presence of indomethacin.[143] It has been reported also that addition of exogenous $PGE_2$ to murine peritoneal macrophages did not alter the ability of these cells to produce high levels of tumour-cell lysis when stimulated with LPS.[144] Finally, contrary to suggestions in Refs. 143 and 144 blockade of prostaglandin synthesis by indomethacin prevented the effect of LPS and led to a substantial resumption of target growth in the presence of activated macrophages.[145] A differential effect of $PGE_2$ on expression of macrophage antitumour activity was described in relation to the state of the macrophages: culture conditions that caused increased $PGE_2$ production by activated macrophages resulted in inhibition of their tumoricidal activity but production of high levels of $PGE_2$ by resident and peptone elicited macrophages was associated with an increase in antitumour activity.[146]

The contradictory results described may be due to differences in sources of macrophages, in types of target tumour cells and in the physiological state of the effector cells.

We and others have investigated the interrelation between eicosanoids and TNF-α or IL-1β in expression of antitumour activity. Human peritoneal macrophages collected from CAPD patients during an intercurrent infectious inflammation showed a sharp drop in cAMP and a decrease in production of cyclooxygenase metabolites.[147] On the other hand, they were primed in an *in vivo* inflammatory environment so that they were much more cytostatic against murine tumour cells than macrophages collected during inflammation free periods.[148] When macrophages collected during inflammation were cultured with LPS, their antitumour cytostasis against two murine tumour-cell lines was markedly increased and this increase was associated with increase in TNF-α and IL-1β release.[34,35,148]

Interrelation between eicosanoids and TNF-α or IL-1β in expression of antitumour activity was also examined by addition of the cell-free compounds to cultures of tumour cells. Interestingly, concomitant addition of $PGE_2$ enhanced the antitumour effect of IL-1β on a IL-1β susceptible WEHI-3B murine tumour, whereas addition of $LTC_4$ inhibited the antitumour effect of IL-1.[149] The synergistic effect between prostaglandins and cytokines in expression of antitumour activity was also observed when the WEHI-3B murine tumour cells were first treated with the cytokine and afterwards with the prostaglandin: pretreatment with IL-1$\beta$ rendered the tumour cells susceptible to $PGE_2$ or $PGI_2$ whereas only susceptibility to $PGE_2$ was increased by pretreatment with TNF-$\alpha$.[123] Other authors found that Kupffer resident rat cells and Kupffer inflammatory murine liver cells produced both TNF-$\alpha$ and $PGE_2$. Upon activation with IFN-$\gamma$ + LPS (for mouse resident Kupffer cells) or with LPS alone (for rat Kupffer cells and mouse inflammatory Kupffer cells) the cells produced more $PGE_2$ and more TNF-$\alpha$.[150] However, $PGE_2$ did not play a role in tumour because treatment with indomethacin increased the TNF induced killing.[150]

### Therapeutic implications

Work on therapeutic effectiveness was mostly concentrated on ways to affect *in vivo* prostaglandin production and on the possibility of using TNF-$\alpha$ either alone or in combination with other agents for therapy. The therapeutic effectiveness was examined in three systems: against animal tumours, against xenogeneic transplants of human tumours in nude mice and in clinical trials in cancer patients.

*Experimental animal tumours:* Prostaglandins have been implicated as enhancers of tumour growth and spread.[93,151] The sources of prostaglandin were tumour cells[99] and/or cells of monocyte-macrophage lineage. Accordingly, it was assumed that inhibition of prostaglandin biosynthesis by cyclooxygenase inhibitors might have a beneficial therapeutic effect.

Most of the work on the basis of this assumption was done by looking on the therapeutic effect of the cyclooxygenase inhibitor IND as downgrading $PGE_2$ production. Thus, it was shown that IND therapy prevents tumour metastasis of a mouse mammary carcinoma[94] cures B16F10 murine melanoma lung metastasis when given in combination with IL-2,[95] and cures murine Ehrlich ascites tumours when administered in combination with LAK cells and IL-2.[96] The effectiveness of IND therapy was correlated to the ability of murine tumours to produce prostaglandin.[99] However, it was doubted if the therapeutic effectiveness of indomethacin is due to inhibition of $PGE_2$ production.[152] IND treatment prolonged survival of sarcoma-bearing mice without, however, having an effect on serum concentrations of IL-6 which increased progressively with increase of the tumour.[153]

Tumour necrosis-like activities have been described since the initial observations in the 1890s on regression of tumours in patients with concomitant bacterial infections or injected with bacterial culture filtrates (for review see Ref. 3). After its first characterization as tumour necrosis factor produced by macrophages,[4] its activity against a long series of human and murine tumours was demonstrated *in vitro*.[111,112] The next obvious step was to determine the therapeutic effectiveness of TNF-$\alpha$ in tumour-bearing hosts. rHuTNF-$\alpha$ was shown to be effective against subcutaneously implanted murine MethA sarcoma but not against the same tumour injected i.p.[154] The curative effect of TNF-$\alpha$ was attributed to its ability to induce local haemorrhages because of its effect on the endothelial cells.[154] It should be noted also that TNF-$\alpha$ therapy involved generation of specific cell-mediated antitumour immunity by a still undefined mechanism.[154]

In another experimental system the antitumour effect of recombinant murine TNF-$\alpha$ given either by continuous i.v. infusion or by repeated i.v. injections was determined in a rat liver metastases model.[155] Only early continuous infusion had an effect on the number of liver metastases presumably because higher doses of TNF-$\alpha$ were tolerated by this schedule.[155] The conclusion of the authors was that TNF-$\alpha$ by itself is not a very efficient antitumour agent and it might be necessary to use TNF-$\alpha$ in combination with other antitumour agents.[155] A similar conclusion of more therapeutic effectiveness of TNF in combination with other treatments was in the case of TNF radiotherapy by comparison with TNF-$\alpha$ alone in rat renal-cell carcinoma,[156] or a combination of TNF-$\alpha$ with the interferon-inducer bropirimine in rat colon cancer.[157] Sequential use of anti-CD3, IL-2 and TNF for LAK induction and maintenance potentiated antitumour activity against a pulmonary metastatic model in mice.[158]

Another interesting additive effect was described after combined treatment with activated macrophages and a low dose of TNF-$\alpha$ in mice bearing Lewis lung carcinoma or EMT6 sarcoma.[159] A synergistic therapeutic effect was also described in tumour-bearing mice treated with low doses of IL-6 in combination with subtherapeutic doses of TNF-$\alpha$.[127] The therapeutic use of TNF-$\alpha$ in tumour-bearing mice was found to be affected by an increase in the toxicity of TNF-$\alpha$ in tumour-bearing hosts[160] and by induction of tolerance to TNF-$\alpha$.[161] A side effect of antibodies to TNF-$\alpha$ was reported: passive immunization with anti-TNF antibodies abrogated partially IL-2 toxicity in tumour-bearing mice.[162] Finally, the effect of TNF therapy might differ in context to the strain of mice: the curative effect of TNF was stronger against MethA sarcomas implanted in BALB/c nu/ + mice than into BALB/c nu/nu mice, when TNF was injected i.v. and similar when injected i.t.[163] A new therapeutic approach was described recently: a novel chimera tumour necrosis factor (TNF-$S_{TH}$) constructed by connecting a modified recombinant human TNF-$\alpha$ (rTNF-S) with thymosin-$\beta_4$ was suggested to be a promising approach for obtaining molecules that more favourably attack tumours than conventional rTNF.[164]

*Xenografts of human tumours in nude mice:* TNF was also found to be effective against human malignant melanoma, human gastric cancer and nasopharyngeal carcinoma cell lines implanted in nude mice.[163] Combined therapy with IFN-$\gamma$ and TNF-$\alpha$ was found to be more effective than TNF-$\alpha$ alone against human ovarian cancer cells inoculated in nude mice.[165] A similar synergistic effect between IFN (interferon-alpha) and TNF-$\alpha$ was described against a human tumour line causing lung metastasis and intra-abdominal carcinomatosis in nude mice.[166] In another study it was found that combined treatment with TNF-$\alpha$ and etoposide was efficient against a human renal cell carcinoma implanted in athymic mice.[167]

*Clinical trials:* The findings on cytotoxic effects of cytokines (especially TNF-$\alpha$) on a wide array of murine and human tumour cell lines *in vitro* and the results on therapeutic effectiveness against murine tumours and against human tumour cells implanted in nude mice prompted the start of clinical trials in various cancer patients. Unfortunately the results in clinical trials were not very spectacular. In one of the first studies a certain improvement due to TNF-$\alpha$ therapy was observed in three out of 18 patients: two cases of lymphoma and one case of Hodgkin's.[168] Febrile reactions and other side effects occurred in most of the patients and they could be prevented by steroids and IND.[168] However, according to the authors, "such prophylaxis may not be desirable because its influence on possible therapeutic benefits is unknown". Some beneficial response in cases of gastric cancer and non-Hodgkin's lymphoma were also reported by other authors.[169] Side effects to TNF-$\alpha$ were recorded and could be ameliorated by IND or ketoprofen.[169] In cases of B-lymphoma some improvement was detected in one out of 26 patients and side effects to TNF-$\alpha$ were again observed.[170] In another study no antitumour response was detected in 29 cancer patients.[171] In two cases out of 16 evaluable patients with disseminated cancer, TNF-$\alpha$ therapy had some antitumour effect: regression of a neck lesion in one case and resolution of malignant ascites in another.[172] The results on various clinical trials with TNF-$\alpha$ until 1988 were summarized at a seminar of the 19th German Cancer Congress: "Although TNF as a single agent is unlikely to be of major benefit for patients with cancer, it has to be concluded that the great expectations which were particularly due to its dramatic effect in the murine sarcoma model leading to the designation 'TNF' have been disappointed".[173]

More clinical trials have been performed with TNF-$\alpha$ in recent years. In a phase II trial with 22 eligible patients with metastatic colorectal adenocarcinoma, treatment with TNF-$\alpha$ was not effective.[174] A more promising result was obtained in a clinical trial with TNF-$\alpha$ including 29 patients with refractory malignant ascites: out of 29 patients, 22 responded with a complete (16) or partial (6) resolution of their ascites.[175] Recently, in one out of 53 patients with advanced malignancies a partial response to TNF was observed in one patient with colorectal carcinoma.[176] In another recent study no clinical efficacy of TNF-$\alpha$ was found in a phase I trial with patients with advanced cancer.[177] Rather disappointing results concerning the use of human recombinant TNF-$\alpha$ for cancer therapy were reported recently by two groups: no objective responses were observed in 22 cases of advanced carcinoma of the pancreas[178] and no significant antitumour activity of rHTNF-$\alpha$ was detected in 127 eligible patients with diverse metastatic malignancies.[179] Another group concluded that rHTNF-$\alpha$ has only modest antitumour activity in 26 patients with renal cell carcinoma.[180]

A more promising approach for therapy was suggested by using TNF-$\alpha$ in combination with other interleukins.[181,182] This was suggested by results obtained *in vitro* and in experimental systems *in vivo* with such combinations. However, in only one patient with melanoma and one patient with mesothelioma (out of 36 patients), was some response observed to combined treatment of recombinant TNF-$\alpha$ with recombinant IFN-$\gamma$,[183] and two partial responses were seen in a study with combined recombinant IL-2 followed by recombinant TNF therapy in 31 patients with metastatic malignancies.[184]

The use of TNF-$\alpha$ therapy is handicapped by the toxicity of the agent and by its extremely pleiotropic biological effects. It should be also noted that in most clinical trails with TNF-$\alpha$ most of the patients were found refractory to other kinds of treatments and were in an advanced stage of disease. Moreover, it was reported that sera of cancer patients may contain factor(s) inhibiting TNF.[185,186] It has been also reported that treatment with TNF-$\alpha$ might induce decreases of NK cell activity and of monocyte production in cancer patients.[187]

The general conclusion from clinical trials until now is that therapy with TNF-$\alpha$ is still in its infancy. Apparently, combinations of various interleukins for cancer therapy might be more promising. A new approach was suggested in the recent years consisting of therapy by monocytes from cancer patients induced to maturate *in vitro* to macrophages

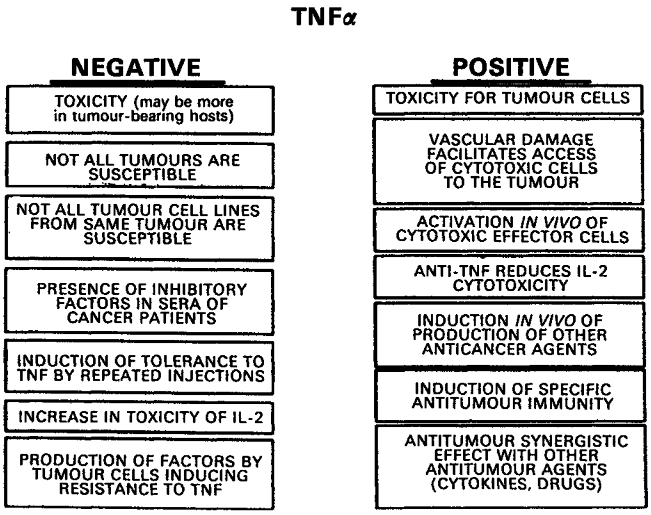

FIG. 3. Negative and positive aspects of the TNFα anticancer therapy.

possessing antitumour cytotoxic activity.[188,189] In view of the findings that such macrophages secret various cytokines,[20] it might be that the activated macrophages continue to secrete *in vivo* interleukins cytotoxic for cancer cells.

### Concluding Remarks

A vaste amount of material has accumulated on the interrelationship in production between macrophages cytokines, macrophage cytokines and eicosanoids, production of these products by tumour cells and the effect of tumour burden on their production. The expression of interrelationship between macrophage cytokines themselves and between macrophage cytokines and eicosanoids was also extremely investigated. In certain instances the results obtained in *in vitro* systems were also expressed *in vivo* in various therapeutic schedules in mice bearing syngeneic tumours on xenografts of human tumour-cell lines. Unfortunately, the promising results obtained *in vitro* and in experimental systems *in vivo* have not yet been well duplicated in clinical trials. Much more work has still to be done in defining optimal conditions for the use of single interleukins and (more likely) combinations of interleukins for effective anticancer therapy. Some of the negative and positive aspects of use of one of the most commonly tested cytokine (TNF-α is schematically represented in Fig. 3). It is also possible that other approaches such as therapy with cells able to produce *in vivo* a wide array of cytokines (macrophages) or devising ways for effective induction of cytotoxic interleukins *in vivo* by a cancer patient's own cells might lead to more promising results.

### References

1. Metchnikoff E. In: *Immunity in Infectious Disease.* Cambridge University Press, London, 1905.
2. Adams DO, Hamilton TA. Phagocytic cells: cytotoxic activities of macrophages. In: Gallin JI, Goldstein IM, Snyderman R, eds. *Inflammation: Basic Principles and Clinical Correlates.* New York: Raven Press, 1988; 471–492.
3. Ruff MR, Gifford GE. Tumor necrosis factor. In: Pick E, Landy M, eds. *Lymphokines.* Academic Press, 1991; **2:** 235–272.
4. Carswell EA, Old LJ, Kassel RL, Green S, Fiore N, Williamson B. An endotoxin-induced serum factor that causes necrosis of tumors. *Proc Nat Acad Sci USA* 1975; **72:** 3666–3670.
5. Gery I, Gershon RK, Waksman BH. Potentiation of the T-lymphocyte response to mitogens. I. The responding cell. *J Exp Med* 1972; **136:** 128–142.
6. Gery I, Waksman BH. Potentiation of the T-lymphocyte response to mitogens. II. The cellular source of potentiating mediator(s). *J Exp Med* 1972; **136:** 143–155.
7. Aarden LA, Brunner TK, Cerottini JC, *et al.* Revised nomenclature for antigen-nonspecific T-cell proliferation and helper factors. *J Immunol* 1979; **123:** 2928–2929.
8. Weissenbach J, Chernajovsky Y, Zeevi M, *et al.* Two interferon mRNAs in human fibroblasts: *in vitro* translation and *Escherichia coli* cloning studies. *Proc Natl Acad Sci USA* 1980; **77:** 7152–7156.
9. Poupart P, Vandenbeele P, Cayphas S, *et al.* B cell growth modulating and differentiating activity of recombinant human 26-kD protein (BSF.2, HuIFN-$B_2$, HPGF). *EMBO J* 1987; **6:** 1219–1224.
10. Aarden LA, De Groot ER, Schaap OL, Lansdorp PM. Production of hybridoma growth factor by human monocytes. *Eur J Immunol* 1987; **17:** 1411–1416.
11. Aggarwal BB. Tumour necrosis factor-TNFα and TNFβ: their structure and pleiotropic biological effects. In: *Drugs and Future* 1987; **12:** 841–848.
12. Dinarello CA. Interleukin 1. *Rev Infect Dis* 1984; **6:** 55–63.
13. Schindler R, Mancilla J, Endres S, Ghorbani R, Clark SC, Dinarello CA. Correlations and interactions in the production of interleukin-6 (IL-6), IL-1, and tumor necrosis factor (TNF) in human blood mononuclear cells: IL-6 suppresses IL-1 and TNF. *Blood* 1990; **75:** 4–47.
14. Sher T, Rottem S, Gallily R. *Mycoplasma capricolum* membranes induce tumor necrosis factor α by a mechanism different from that of lipopolysaccharide. *Cancer Immunol Immunother* 1990; **31:** 86–92.
15. Riesenfeld-Orn I, Wolpe S, Garcia-Bustos JF, Hoffmann MK, Tuomanen E. Production of interleukin-1 but not tumor necrosis factor by human monocytes stimulated with pneumococcal cell surface components. *Inf Immun* 1989; **57:** 1890–1893.
16. Kovacs EJ, Radzioch D, Young HA, Varesio L. Differential inhibition of IL-1 and TNFα mRNA expression by agents which block second messenger pathways in murine macrophages. *J Immunol* 1988; **141:** 3101–3105.
17. Burchett SK, Weaver WM, Westall JA, Larsen A, Kronheim S, Wilson CB. Regulation of tumor necrosis factor/cachectin and IL-1 secretion in human mononuclear phagocytes. *J Immunol* 1988; **140:** 3473–3481.
18. Chensue SW, Shmyr-Forsch C, Weng A, Otterness IG, Kunkel SL. Biologic and immunohistochemical analysis of macrophage interleukin-1, -1β, and tumor necrosis factor production during the peritoneal exudative response. *J Leuk Biol* 1989; **46:** 529–537.

19. Becker S, Devlin RB, Haskill JS. Differential production of tumor necrosis factor, macrophage colony stimulating factor, and interleukin-1 by human alveolar macrophages. *J Leuk Biol* 1989; **45**: 353–361.
20. Scheiberbogen C, Andreesen R. Developmental regulation of the cytokine repertoire in human macrophages: IL-1, IL-6, TNFα, and M-CSF. *J Leuk Biol* 1991; **50**: 35–42.
21. Nawroth PP, Bank I, Handley D, Cassimeris J, Chess L, Stern D. Tumor necrosis factor/cachectin interacts with endothelial cell receptors to induce release of interleukin-1. *J Exp Med* 1986; **163**: 1363–1375.
22. Bachwich PR, Chensue SW, Larrick JW, Kunkel SL. Tumor necrosis factor stimulates interleukin-1 and prostaglandin $E_2$ production in resting macrophages. *Biochem Biophysiol Res Com* 1986; **136**: 94–101.
23. Dinarello CA, Cannon JG, Wolff SM, *et al.* Tumor necrosis factor/cachectin is an endogenous pyrogen and induces production of interleukin-1. *J Exp Med* 1986; **163**: 1433–1450.
24. Shalaby MR, Waage A, Aarden L, Espevik T. Endotoxin, tumor necrosis factor and interleukin 1 induce interleukin 6 production *in vivo*. *Clin Immunol Immunopathol* 1989; **53**: 488–498.
25. Bauer J, Bauer TM, Kalb T, *et al.* Regulation of interleukin 6 receptor expression in human monocytes and monocyte-derived macrophages. Comparison with the expression in human hepatocytes. *J Exp Med* 1989; **170**: 1537–1549.
26. Aderka D, Le JM, Vilcek J. IL-6 inhibits lipopolysaccharide-induced tumor necrosis factor production in cultured human monocytes, U937 cells, and in mice. *J Immunol* 1989; **143**: 3517–3523.
27. Hauschildt S, Hoffmann P, Beutscher HU, *et al.* Activation of bone marrow derived mouse macrophages by bacterial lipopeptide: cytokine production, phagocytosis and Ia expression. *Eur J Immunol* 1990; **20**: 63–68.
28. Bauer J, Ganter U, Geiger T, *et al.* Regulation of interleukin 6 expression in cultured human blood monocytes and monocyte-derived macrophages. *Blood* 1988; **72**: 1134.
29. Kotloff RM, Little J, Elias JA. Human alveolar macrophage and blood monocyte interleukin 6 production. *Am J Respir Cell Mol Biol* 1990; **3**: 497–505.
30. Roztoczy I, Content J. The effects of various cytokines on interleukin 6 and interfon α synthesis in human peripheral blood mononuclear cells. *J Interferon Res* 1990; **10**: 637–645.
31. Martin CA, Dorf ME. Interleukin 6 production by murine macrophage cell lines P388D1 and J774A: stimulation requirements and kinetics. *Cell Immunol* 1990; **128**: 555–568.
32. Lepe-Zuniga JL, Gery I. Production of intra- and extracellular interleukin-1 (IL-1) by human monocytes. *Clin Immunol Immunopathol* 1984; **31**: 222–230.
33. Aznar C, Fitting C, Cavaillon JM. Lipopolysaccharide-induced production of cytokines by bone marrow-derived macrophages: dissociation between intracellular interleukin 1 production and interleukin 1 release. *Cytokine* 1990; **2**: 259–265.
34. Fieren MWJA, Bemd van den GJCM, Bonta IL, Ben-Efraim S. Peritoneal macrophages from patients on continuous ambulatory peritoneal dialysis have an increased capability to release tumor necrosis factor during peritonitis. *J Clin Lab Immunol* 1991; **34**: 1–9.
35. Fieren MWJA, Bemd van den GJCM, Bonta IL. Endotoxin-stimulated peritoneal macrophages obtained from continuous ambulatory peritoneal dialysis patients show an increased capacity to release interleukin-1β *in vitro* during infectious peritonitis. *Eur J Clin Invest* 1990; **20**: 453–457.
36. Kunkel SL, Spengler M, May MA, Spengler R, Larrick J, Remick D. Prostaglandin $E_2$ regulates macrophage-derived tumor necrosis factor gene expression. *J Biol Chem* 1988; **263**: 5380–5384.
37. Karck U, Peters T, Decker K. The release of tumor necrosis factor from endotoxin-stimulated rat Kupfer cells is regulated by prostaglandin $E_2$ and dexamethasone. *J Hepatol* 1988; **7**: 352–361.
38. Spatafora M, Chiappara G, D'Amico D, *et al.* Prostaglandin $E_2$ down-regulates the expression of tumor necrosis alpha gene by human blood monocytes. In: Samuelsson B, Ed. *Adv Prostagl Thromb Leuk Res.* New York: Raven Press, 1990; **21**: 521–524.
39. Renz H, Gong JH, Schmidt A, Nain M, Gemsa D. Release of tumor necrosis factor α from macrophages. Enhancement and suppression are dose-dependently regulated by prostaglandin $E_2$ and cyclic nucleotides. *J Immunol* 1988; **141**: 2388–2393.
40. Gong JH, Renz H, Sprenger H, Naim M, Gemsa D. Enhancement of tumor necrosis factor α gene expression by low doses of prostaglandin $E_2$ and cyclic GMP. *Immunobiol* 1990; **182**: 44–55.
41. Spengler RN, Spengler ML, Lincoln P, Remick DG, Strieter RM, Kunkel SL. Dynamics of dibutyryl cyclic AMP- and Prostaglandin $E_2$-mediated suppression of lipopolysaccharide-induced tumor necrosis factor alpha gene expression. *Inf Immun* 1989; **57**: 2837–2841.
42. Fieren MWJA, Bemd van den GJCM, Ben-Efraim S, Bonta IL. Prostaglandin $E_2$ inhibits the release of tumor necrosis factor-α rather than interleukin 1β, from human macrophages. *Immunol Letters* 1991; **31**: 85–90.
43. Gagnon L, Fillion LG, Dubois C, Rola-Pleszczynski M. Leukotrienes and macrophage activation: augmented cytotoxic activity and enhanced interleukin 1, tumor necrosis factor and hydrogen peroxide production. *Agents and Actions* 1989; **26**: 141–147.
44. Goetzl EJ, Hill HR, Gorman RR. Unique aspects of the modulation of human neutrophil function by 12-L-hydroperoxy-5,8,10-eicosatetraenoic acid. *Prostaglandins* 1980; **19**: 71–85.
45. Schade UF, Ernst M, Reinke M, Wolter DT. Lipoxygenase inhibitors suppress formation of tumor necrosis factor *in vitro* and *in vivo*. *Biochem Biophys Res Comm* 1989; **159**: 748–754.
46. Schade UF, Engel R, Jakobs D. The role of lipoxygenases in endotoxin-induced cytokine production. *Prog Clin Biol Res* 1991; **367**: 73–82.
47. Hoffman T, Lee YL, Lizzio EF, *et al.* Absence of modulation of monokine production via endogenous cyclooxygenase or 5-lipoxygenase metabolites: MK-886(3-(4-chlorobenzyl)-3-t-butyl-thio-5-isopropylindol-2-yl)-2,2-dimethylpropanoic acid), indomethacin, or arachidonate fail to alter immunoreactive interleukin-1β, or TNFα production by human monocytes *in vitro*. *Clin Immunol Immunopathol* 1991; **58**: 399–408.
48. Hart PH, Whitty GA, Piccoli DS, Hamilton JA. Control by IFN-τ and $PGE_2$ of TNFα and IL-1 production by human monocytes. *Immunology* 1983; **130**: 890–895.
49. Dinarello CA, Bishai I, Rosenwasser IJ, Coceani F. The influence of lipoxygenase inhibitors on the *in vitro* production of human leukocytic pyrogen and lymphocyte activating factor (interleukin-1). *Int J Immunopharmac* 1984; **6**: 43–50.
50. Farrar W, Humes JL. The role of arachidonic acid metabolism in the activities of interleukin 1 and 2. *J Immunol* 1985; **135**: 1153–1159.
51. Rola-Pleszczynski M, Lemaire I. Leukotrienes augment interleukin 1 production by human monocytes. *J Immunol* 1985; **135**: 3958–3961.
52. Rola-Pleszczynski M, Stankova J. Cytokine gene regulation by $PGE_2$, $LTB_4$ and PAF. *Mediators of Inflammation* 1992; **1**: 5–8.
53. Kunkel SL, Chensue SW, Phan SH. Prostaglandins as endogenous mediators of interleukin 1 production. *J Immunol* 1986; **136**: 186–192.
54. Knudsen PJ, Dinarello CA, Strom TB. Prostaglandin post-transcriptionally inhibit monocyte expression of interleukin-1 activity by increasing cyclic adenosine monophosphate. *J Immunol* 1986; **137**: 3187–3194.
55. Mugridge KG, Perretti M, Parente L. 5-Lipoxygenase activation may facilitate an interleukin-1 transduction signal. In: Samuelsson B, ed. *Adv Prostagl Thromb Leuk Res.* New York: Raven Press, 1990; **21**: 517–520.
56. Dinarello CA. The biology of interleukin-1 and comparison to tumour necrosis factor. *Immunol Lett* 1987; **16**: 227–231.
57. Otterness IG, Bliven ML, Eskra JD, Reinke M, Hanson DC. The pharmacologic regulation of interleukin-1 production: the role of prostaglandins. *Cell Immunol* 1988; **114**: 385–397.
58. Scales WE, Chensue SW, Otterness I, Kunkel SL. Regulation of monokine gene expression: prostaglandin $E_2$ suppresses tumor necrosis factor but not interleukin-1α of β-mRNA and cell-associated bioactivity. *J Leuk Biol* 1989; **45**: 416–421.
59. Fieren MWJA, Bemd van den GJCM, Bonta IL. Peritoneal macrophages from patients on continuous ambulatory peritoneal dialysis show a differential secretion of prostanoids and interleukin-1β. *Prostagl Leuk Ess Fatty Acids* (in press).
60. Callery MP, Mangino MJ, Kamei T, Fye MW. Interleukin-6 production by endotoxin-stimulated Kupfer cells is regulated by prostaglandin $E_2$. *J Surgic Res* 1990; **48**: 523–527.
61. Komatsu H, Yahu H, Chiba K, Okumoto T. Inhibition by cyclooxygenase inhibitors of interleukin-6 production by human peripheral blood mononuclear cells. *Int J Immunopharmac* 1990; **13**: 1137–1146.
62. Poubelle P, Stankova J, Grassi J. Leukotriene $B_4$ up-regulates IL-6 rather than IL-1 synthesis in human monocytes. *Agents and Actions* 1991; **34**: 42–45.
63. Stankova J, Rola-Pleszczynski M. Interleukin-6 production by mononuclear phagocytes can be stimulated by leukotrienes. *Arch Immunol Therap Exp* 1992; (in press).
64. Khansari N, Chou YK, Fudenberg HH. Human monocyte heterogeneity: interleukin 1 and prostaglandin $E_2$ production by separate subsets. *Eur J Immunol* 1985; **15**: 48–51.
65. Mantovani A, Ming WJ, Balotta C, Abdeljalil B, Botazzi B. Origin and regulation of tumor-associated macrophages: the role of tumor-derived chemotactic factor. *Biochim Biophys Acta* 1986; **865**: 59–67.
66. McBride WH. Phenotype and functions of intratumoral macrophages. *Biochim Biophys Acta* 1986; **865**: 27–41.
67. Vincent JE, Vermeer MA, Kort WJ, Zijlstra FJ. The formation of thromboxane $B_2$, leukotriene $B_4$ and 12-hydroxysatetraenoic acid by alveolar macrophages after activation during tumor growth in the rat. *Biochinm Biophys Acta* 1990; **1042**: 255–258.
68. Korst WJ, Zijlstra FJ, Weijma IM. Eicosanoid synthesis by alveolar macrophages in rats with malignant mammary tumors: differences in rats treated with and without carrageenan implants. *Prostagl Leuk Ess Fatty Acids* 1989; **37**: 113–120.
69. Hiodaka T, Tsurata M, Tomita Y, Inokuchi T, Sugiyama M, Ogura R. Generation of leukotrienes and hydroxyeicosatetraenoic acids in peritoneal macrophages of tumor-bearing mice. *Prostagl Leuk Ess Fatty Acids* 1991; **44**: 185–190.
70. Parhar RS, Lala PK. Prostaglandin $E_2$-mediated inactivation of various killer lineage cells by tumor-bearing host macrophages. *J Leuk Biol* 1988; **44**: 474–484.
71. Plescia OJ, Pontieri GM, Brown J, *et al.* Amplification by macrophages of prostaglandin-mediated immunosuppression in mice bearing syngeneic tumors. *Prostagl Leuk & Med* 1984; **16**: 205–223.
72. Young MR, Hoover CS. Inhibition of spleen cell cytotoxic capacity toward tumor by elevated prostaglandin $E_2$ levels in mice bearing Lewis lung carcinoma. *J Natl Cancer Inst* 1986; **77**: 425–429.
73. Palleroni AV, Varesio L, Wright RB, Brunda MJ. Tumoricidal alveolar macrophage and tumor infiltrating macrophage cell lines. *Int J Cancer* 1991; **49:** 296–302.
74. Moldawer LL, Lonnroth C, Mizel SB, Lundholm KG. Down-regulation of interleukin-1 production by macrophages of sarcoma-bearing mice. *J Immunol* 1987; **138:** 4270–4272.
75. Brunda MJ, Sulich V, Wright RB, Palleroni AV. Tumoricidal activity and cytokine secretion by tumor-infiltrating macrophages. *Int J Cancer* 1991; **48:** 704–708.
76. Peri G, Rossi V, Taraboletti A, Erroi A, Mantovani A. Ia antigen expression and IL-1 activity in murine tumor-associated macrophages. *Immunology* 1986; **59:** 527–533.
77. Wilson KM, Lord EM. Specific (EMT6) and non-specific (WEHI-164) cytolytic activity by host cells in filtrating tumor spheroids. *Br J Cancer* 1987; **55:** 141–146.
78. Wilson KM, Siegal G, Lord EM. Tumor necrosis factor-mediated cytotoxicity by tumor-associated macrophages. *Cell Immunol* 1989; **123:** 158–165.
79. Mannel DN, Janicke R, Westenfelder U, Echtenacher B, Kist A, Falk N. Tumor-induced tumor necrosis factor production in macrophages. *Lymphokine Res* 1990; **9:** 485–489.
80. Cameron DJ, Rittenbury M, Majeski J. Ability of cancer patient's macrophages to kill autologous tumor targets. Effects of prostaglandin inhibitors on cytotoxicity. *Cancer* 1984; **53:** 2053–2057.
81. Cameron DJ, Stromberg BV. The abilty of macrophages from head and neck cancer patients to kill tumor cells. *Cancer* 1984; **54:** 2403–2408.
82. Sébahoun G, Maraninchi D, Carcassonne Y. Increased prostaglandin $E_2$ production in maligant lymphomas. *Acta Hemat* 1985; **74:** 132–136.
83. Economou JS, Colquhon SD, Anderson TM, *et al.* Interleukin-1 and tumor necrosis factor production by tumor-associated mononuclear leukocytes and peripheral mononuclear leukocytes in cancer patients. *Int J Cancer* 1988; **42:** 712–714.
84. Erroi A, Sironi M, Chiaffarino F, Chen ZG, Mengozzi M, Mantovani A. IL-1 and IL-6 release by tumor associated macrophages from human ovarian carcinoma. *Int J Cancer* 1989; **44:** 795–801.
85. Zielinski CC, Mueller C, Tyl E, Tichatschek E, Kubista E, Spona J. Impaired production of tumor necrosis factor in breast cancer. *Cancer* 1990; **66:** 1944–1948.
86. Barra BP, Rogers LR, Thomassen MJ, Barnett GH, Estes ML. Monocyte tumoricidal activity and tumor necrosis factor production in patients with malignant brain tumors. *Cancer Immunol Immunother* 1991; **33:** 314–318.
87. Siziopikou KP, Harris JE, Casey L, Nawas Y, Braunm DP. Impaired tumoricidal function of alveolar macrophages from patients with non-small cell lung cancer. *Cancer* 1991; **68:** 1035–1044.
88. Numata A, Minagawa T, Asano M, Nakane, Katoh H, Tanabe T. Functional evaluation of tumor-infiltrating mononuclear cells. Detection of endogenous interferon-τ and tumor necrosis factor-α in human colorectal adenocarcinoma. *Cancer* 1991; **68:** 1937–1943.
89. Nara K, Odagiri H, Fujii M, *et al.* Increased production of tumor necrosis factor and prostaglandin $E_2$ by monocytes in cancer patients and its unique modulation by their plasma. *Cancer Immunol Immunother* 1987; **25:** 126–132.
90. Ikemoto S, Kishimoto T, Nishio S, Wada S, Maekawa M. Correlation of tumor necrosis factor and prostaglandin $E_2$ production of monocytes in bladder cancer patients. *Cancer* 1989; **64:** 2076–2080.
91. Malick AP, Elgert KD, Garner RE, Adkinson NG. Prostaglandin $E_2$ production by Mac-$2^+$ macrophages: tumor-induced shift. *J Leuk Biol* 1987; **42:** 673–681.
92. Janicke R, Mannel DN. Distinct tumor cell membrane constituents activate human monocytes for tumor necrosis factor synthesis. *J Immunol* 1990; **144:** 1144–1150.
93. Rolland PH, Martin PM, Jacquemier J, Rolland AM, Toga M. Prostaglandin in human breast cancer: evidence suggesting that an elevated prostaglandin production is a marker of high metastatic potential for neoplastic cells. *J Natl Cancer Inst* 1980; **64:** 1061–1070.
94. Lala PK, Parhar RS, Singh P. Indomethacin therapy abrogates the prostaglandin-mediated suppression of natural killer activity in tumor-bearing mice and prevents tumor metastasis. *Cell Immunol* 1986; **99:** 108–118.
95. Lala PK, Parhar RS. Cure of B16F10 melanoma lung metastasis in mice by chronic indomethacin therapy combined with repeated rounds of interleukin-2: characteristics of killer cells generated *in situ*. *Cancer Res* 1988; **48:** 1072–1079.
96. Lala PK, Parhar PS, Singh P, Lala PK. Cure of murine Ehrlich ascites tumors with chronic oral indomethacin therapy combined with intraperitoneal administration of LAK cells and IL-2. *Cancer Letters* 1990; **51:** 27–35.
97. Lala PK, Elkashab M, Kerbel RS, Parhar RS. Cure of human melanoma lung metastases in nude mice with chronic indomethacin therapy combined with multiple rounds of IL-2: characteristics of killer cells generated *in situ*. *Intern Immunol* 1990; **2:** 1149–1158.
98. Sykes JAC, Maddox IS. Prostaglandin production by experimental tumors and effects of anti-inflammatory compounds. *Nature New Biology* 1972; **237:** 59–61.
99. Furuta Y, Hall ER, Sanduka S, Barkley T Jr., Milas L. Prostaglandin production by murine tumors as predictor for therapeutic response to indomethacin. *Cancer Res* 1988; **48:** 3002–3007.
100. Mahan M, Meunier JA, Newby M, Young MR. Prostaglandin $E_2$ production by EL4 leukemia cells from C57BL/6 mice: mechanism for tumor dissemination. *J Natl Cancer Inst* 1985; **74:** 191–195.
101. Hubbard WC, Alley MC, McLemore TL, Boyd MR. Profiles of prostaglandin synthesis in sixteen established cell lines derived from human lung, colon, prostate and ovarian tumors. *Cancer Res* 1988; **48:** 4770–4775.
102. Nakazawa I, Ohuchi K, Watanabe M, Tsurufuji S. A difference in prostaglandin $E_2$ synthesis between cancer cells metastasizing into liver and kidney. *Prostagl Leuk Med* 1985; **17:** 265–266.
103. Lönnroth Chr, Moldddawer LL, Gelin J, Kindblom L, Sherry B, Lundholm K. Tumor necrosis factor-α and interleukin-1α production in cachectic, tumor-bearing mice. *Int J Cancer* 1990; **46:** 889–896.
104. Kobari L, Weil D, Lemoine FM, *et al.* Secretion of tumor necrosis factor-α by fresh acute nonlymphoblastic leukemic cells: role in the disappearance of normal CF*U-GM progenitors. *Exp Hematol* 1990; **18:** 1187–1192.
105. Hata H, Matsuzaki H, Takatsuki K. Autocrine growth by two cytokines, interleukin-6 and tumor necrosis factor-α, in the myeloma cell line KHM-1A. *Acta Haematol* 1990; **83:** 133–136.
106. Utsumi K, Takai Y, Tada T, Ohzeki S, Fujiwara H, Hamaoka T. Enhanced production of IL-6 in tumor-bearing mice and determination of cells responsible for its augmented production. *J Immunol* 1990; **145:** 397–403.
107. Schoot van der CE, Jansen P, Poorter M, *et al.* Interleukin-6 and interleukin-1 production in acute leukemia with monocytoid differentiation. *Blood* 1989; **74:** 2081–2087.
108. Siegall CB, Schwab G, Nordan RP, FitzGerald DJ, Pastan I. Expression of the interleukin 6 receptor and interleukin 6 in prostate carcinoma cells. *Cancer Res* 1990; **50:** 7786–7788.
109. Partridge M, Chantry D, Turner M, Feldmann M. Production of interleukin-1 and interleukin-6 by human keratinocytes and squamous cell carcinoma cell lines. *J Invest Dermatol* 1991; **96:** 771–776.
110. Ohro I, Tanno Y, Yamauchi K, Takishima T. Production of tumor necrosis factor by mastocytoma P815 cells. *Immunology* 1990; **69:** 312–315.
111. Sugarman BJ, Aggarwal BB, Hass PE, Figari IS, Palladino MA Jr, Shepard HM. Recombinant human tumor necrosis factor-α: effects on proliferation of normal and transformed cells *in vitro*. *Science* 1985; **230:** 943–945.
112. Shepard HM, Lewis GD. Resistance of tumor cells to tumor necrosis factor. *J Clin Immunol* 1988; **8:** 333–341.
113. Morikawa K, Fidler IJ. Heterogeneous response of human colon cancer cells to the cytostatic and cytotoxic effects of recombinant human cytokines: interferon-α, interferon-τ, tumor necrosis factor, and interleukin-1. *J Biol Resp Modifiers* 1989; **8:** 206–218.
114. Heicappell R, Naito S, Ichinose Y, Creasey AA, Lin LS, Fidler IJ. Cytostatic and cytolytic effects of human recombinant tumor necrosis factor on human renal cell carcinoma cell lines derived from a single surgical specimen. *J Immunol* 1987; **138:** 1634–1640.
115. Decker T, Lohmann-Matthes ML, Gifford GE. Cell-associated tumor necrosis factor (TNF) as a killing mechanism of activated cytotoxic macrophages. *J Immunol* 1987; **138:** 957–962.
116. Philip R, Epstein LB. Tumor necrosis factor as immunomodulator and mediator of monocyte cytotoxicity induced by itself, τ-interferon and interleukin-1. *Nature* 1986; **323:** 84–86.
117. Danis M, Ghadirian E. Human monocytes tumouristatic ability: modulation by cytokines and tumour cell products. *Int J Immunopharmac* 1990; **12:** 509–513.
118. Onozaki K, Matsushima K, Aggarwal BB, Oppenheim JJ. Human interleukin-1 is a cytocidal factor for several tumor cell lines. *J Immunol* 1985; **135:** 3962–3968.
119. Cozzolino F, Rubartelli A, Aldinucci D, *et al.* Interleukin 1 as an autocrine growth factor for acute myeloid leukemia cells. *Proc Natl Acad Sci USA* 1989; **86:** 2369–2373.
120. Ruggiero V, Baglioni C. Synergistic anti-proliferative activity of interleukin 1 and tumor necrosis factor. *J Immunol* 1987; **138:** 661–663.
121. Ichinose Y, Tsao JY, Fidler IJ. Destruction of tumor cells by monokines released from activated human blood monocytes: evidence for parallel and additive effects of IL-1 and TNF. *Cancer Immunol Immunother* 1988; **27:** 7–12.
122. Smith DM, Lackides GA, Epstein LB. Coordinated induction of autocrine tumor necrosis factor and interleukin 1 in normal human monocytes and the implications for monocyte-mediated cytotoxicity. *Cancer Res* 1990; **50:** 3146–3153.
123. Ben-Efraim S, Tak C, Bonta IL. Macrophage cytokines render WEHI-3B tumor cells susceptible to cytostasis by prostaglandins. *Prostagl Leuk Ess Fatty Acids* 1990; **40:** 163–167.
124. Ichinose Y, Bakouche O, Tsao JY, Fidler IJ. Tumor necrosis factor and IL-1 associated with plasma membranes of activated human monocytes lyse monokine-sensitive but not monokine-resistant tumor cells whereas viable activated monocytes lyse both. *J Immunol* 1988: **141:** 512–518.
125. Lepoivre M, Boudbid H, Lemaire GH, Petit JF. Cytostatic product(s) released by activated macrophages, unrelated to interleukin 1, tumor necrosis factor-α, and interferon-α/β. *Cell Immunol* 1988; **115:** 273–287.
126. Chen L, Mory Y, Zilberstein A, Revel M. Growth inhibition of human breast carcinoma and leukemia/lymphoma cell lines by recombinant interferon-$\beta_2$. *Proc Natl Acad Sci USA* 1988; **85:** 8037–8041.
127. Mule JJ, McIntosh JK, Jablons DM, Rosenberg SA. Antitumor activity of recombinant interleukin 6 in mice. *J Exp Med* 1990; **171:** 629–636.
128. Kawano M, Hirano T, Matsuda T, *et al.* Autocrine generation and requirement of BSF-2/IL-6 for human multiple myeloma. *Nature* 1988; **332:** 83–85.

129. Miki S, Iwano M, Miki Y, *et al.* Interleukin-6 (IL-6) functions as an *in vitro* autocrine growth factor in renal cell carcinoma. *FEBS Letters* 1989; **250:** 607–610.
130. Jablons DM, McIntosh JK, Mule JJ, Nordan RP, Rudikoff S, Lotze MT. Induction of interferon-$\beta_2$/interleukin-6 (IL-6) by cytokine administration and detection of circulating interleukin-6 in the tumor-bearing state. *Ann NY Acad Sci* 1989; **557:** 157–160.
131. Helle M, Brakenhoff JPJ, De Groot ER, Aarden LA. Interleukin-6 is involved in interleukin-1 induced activities. *Eur J Immunol* 1988; **18:** 957–959.
132. Bonta IL, Ben-Efraim S. Leukotrienes and prostaglandins mutually govern the antitumor potential of macrophages. In: Garaci E, Paoletti R, Santoro MD, eds. *Prostaglandins in Cancer Research.* Berlin: Springer Verlag, 1987; 193–201.
133. Ophir R, Ben-Efraim S, Bonta IL. Leukotriene $D_4$ and indomethacin enhance additively the macrophage cytostatic activity *in vitro. Int J Tissue React* 1987; **9:** 189–194.
134. Bonta IL, Elliott GR, Tak C, Ben-Efraim S. Indomethacin stimulation of macrophage cytostasis against MOPC-315 tumor cells is enhanced by endogenous metabolites of lipoxygenase and counteracted by prostaglandin $E_2$. *Agents and Actions* 1989; **26:** 167–169.
135. Hilten van JA, Elliott GR, Bonta IL. Specific lipoxygenase inhibition reverses macrophage cytostasis towards P815 tumor cells *in vitro* induced by the calcium ionophore A23187. *Prostag1 Leuk Ess Fatty Acids* 1988; **34:** 187–192.
136. Hilten van JA, Ben-Efraim S, Zijlstra FJ, Bonta IL. Leukotriene $C_4$ is an essential 5-lipoxygenase intermediate in A23187-induced macrophage antitumor cytostatic activity against P815 cells. *Prostagl Leuk Ess Fatty Acids* 1990; **39:** 283–290.
137. Hilten van JA. Modulation of macrophage antitumor cytostasis by endogenous leukotrienes. PhD Thesis Erasmus University Rotterdam, 1990.
138. Taffet SM, Eurell TE, Russell SW. Regulation of macrophage-mediated tumor cell killing by prostaglandins: comparison of the effects of $PGE_2$ and $PGI_2$. *Prostaglandins* 1982; **24:** 763–773.
139. Santoro MG, Philpott GW, Jaffe BM. Inhibition of tumor growth *in vivo* and *in vitro* by prostaglandin E. *Nature* 1979; **263:** 777–779.
140. Santoro MG, Benedetto A, Jaffe BM. Prostaglandin $A_1$ induces differentiation in Friend erythroleukemia cells. *Prostaglandins* 1979; **17:** 719–727.
141. Favalli C, Garaci E, Santoro MG, Santucci L, Jaffe BM. The effect of $PGA_1$ on the immunoresponse in melanoma-bearing mice. *Prostaglandins* 1980; **19:** 897–904.
142. Honn KV, Davidowicz K, Bienowski M, Morgan LR, Marnett LJ. Effects of prostaglandin $A_1$ on Harding-Passey melanoma macromolecular synthesis. *Abstracts of 4th International Prostaglandin Conference*, 27–31 May, 1979, Washington DC; 50.
143. Raddasi K, Tenu JP, Pradelles P, Lemaire G. Arachidonate metabolism triggered in primed macrophages by signals including antitumor activity. *J Lipid Med* 1991; **4:** 185–198.
144. Utsugi T, Fidler IJ. Prostaglandin $E_2$ does not inhibit tumoricidal activity of mouse macrophages against adherent tumor cells. *J Immunol* 1991; **146:** 2066–2071.
145. Drapier JC, Petit JF. Development of antitumor activity in LPS-stimulated mouse granuloma macrophages. Regulation by eicosanoids. *Inflammation* 1986; **10:** 195–204.
146. Snider ME, Fertel RH, Zwilling BS. Prostaglandin regulation of macrophage function: endogenous and exogenous prostaglandins. *Cell Immunol* 1982; **74:** 234–242.
147. Adolfs MJP, Fieren MWJA, Bonta IL. Infectious-inflammatory changes in cyclic AMP levels and in their regulation by prostaglandins in human peritoneal macrophages. *Prostagl Leuk Med* 1985; **18:** 217–226.
148. Ben-Efraim S, Tak C, Fieren MJWA, Bemd van den GJCM, Bonta IL. Inflammation amplifies antitumor cytostasis by human peritoneal macrophages. *Med Oncol & Tumor Pharmacother* 1991; **8:** 87–94.
149. Elliott GR, Tak C, Bonta IL. Prostaglandin $E_2$ enhances, and leukotriene $C_4$ inhibits, interleukin-1 inhibition of WEHI-3B cell growth. *Cancer Immunol Immunother* 1989; **27:** 133–136.
150. Decker T, Lohmann-Matthes ML, Karck U, Peters T, Decker K. Comparative study of cytotoxicity, tumor necrosis factor, and prostaglandin release after stimulation of rat Kupfer cells, murine Kupfer cells, and murine inflammatory liver macrophages. *J Leuk Biol* 1989; **45:** 139–146.
151. Honn KV, Bockman RS, Marnett LJ. Prostaglandins and cancer: a review of tumor initiation through tumor metastasis. *Prostaglandins* 1981; **21:** 833–864.
152. Maca RD. Inhibition of the growth of Lewis lung carcinoma by indomethacin in conventional, nude and beige mice. *J Biol Resp Modifiers* 1988; **7:** 568–580.
153. Gelin J, Moldawer LL, Lonnroth C, *et al.* Appearance of hybridoma factor/interleukin-6 in the serum of mice bearing a methylcholantrene-induced sarcoma. *Biochem Biophys Res Commun* 1988; **157:** 575–579.
154. Palladino Ma Jr, Shalaby MR, Kramer SM, *et al.* Characterization of the antitumor activities of human tumor necrosis factor and the comparison with other cytokines. Induction of tumor-specific immunity. *J Immunol* 1987; **138:** 4023–4030.
155. Scheringa M, Keizer A, Jeekel J, Marquet RL. Anti-tumor effect of recombinant murine TNF-α (rMU TNFα) given by continuous i.v. infusion as compared to repeated i.v. injections in a rat liver metasis model. *Int J Cancer* 1989; **43:** 905–909.
156. Moorselaar van RJA, Schachöfer JHM, Crooijmans RPMA, Stratum van P, Debruyne FMJ, Schalken JA. Combined effects of tumor necrosis factor alpha and radiation in the treatment of renal cell carcinoma grown as radia spheroids. *Anticancer Res* 1990; **10:** 1769–1774.
157. Marquet RL, de Bruim RW, Eggermont AM, Jeekel J. Treatment of colon cancer in rats with rMuTNF and the interferon-inducer bropirimine. *Agents and Actions* 1989; **26:** 151–152.
158. Yang SC, Fry KD, Grimm EA, Roth JA. Phenotype and cytolytic activity of mouse tumor-bearer splenocytes and tumor-infiltrating lymphocytes from K-1735 melanoma metastases following anti-CD3, interleukin-2, and tumor necrosis factor-α combination immunotherapy. *J Immunother* 1991; **10:** 362–365.
159. Chokri M, Freudenberg M, Galanos C, Poindron P, Bartholenys J. Antitumoral effects of lipopolysaccharides, tumor necrosis factor, interferon and activated macrophages: synergism and tissue distribution. *Anticancer Res* 1989; **9:** 1185–1190.
160. Krosnick JA, McIntosh JK, Mulé JJ, Rosenberg SA. Studies of the mechanisms of toxicity of the administration of recombinant tumor necrosis factor-α in normal and tumor-bearing mice. *Cancer Immunol Immunother* 1989; **30:** 133–138.
161. Fraker DL, Sheppard BC, Norton JA. Impact of tolerance on a antitumor efficacy of tumor necrosis factor in mice. *Cancer Res* 1990; **50:** 2261–2267.
162. Fraker DL, Langstein HN, Norton JA. Passive immunization against tumor necrosis factor partially abrogates interleukin-2 toxicity. *J Exp Med* 1989; **170:** 1015–1020.
163. Haranaka K, Satomi N, Sakurai A. Antitumor activity of murine tumor necrosis factor (TNF) against transplanted murine tumors and hetero-transplanted human tumors in nude mice. *Int J Cancer* 1984; **34:** 263–267.
164. Noguchi K, Inagawa H, Tsuji Y, Morikawa A, Mizuno DI, Soma GI. Antitumor activity of a novel chimera tumor necrosis factor (TNF-$S_{TH}$) constructed by connecting rTNF-S with thymosin $\beta_4$ against murine syngeneic tumors. *J Immunother* 1991; **10:** 105–111.
165. Balkwill FR, Ward BG, Fiers W. Therapeutic potential of tumor necrosis factor and interferon in experimental human ovarian cancer. *Cancer Res* 1987; **47:** 4755–4758.
166. Naomoto Y, Tanaka N, Orita K. Antitumor effect of natural human tumor necrosis factor-α and natural human interferon-α in combination against human cancer transplanted into nude mice. *Acta Med Okayama* 1989; **43:** 211–221.
167. Donaldson JT, Keane TE, Poulton SH, Walther PJ. Enhanced *in vivo* cytotoxicity of recombinant human tumor necrosis factor with etoposide in human renal cell carcinoma. *Urol Res* 1990; **18:** 245–250.
168. Selby P, Hobbs S, Viner C, *et al.* Tumor necrosis factor in man: clinical and biological observations. *Br J Cancer* 1987; **56:** 803–808.
169. Taguchi T. Clinical studies of recombinant human tumor necrosis factor. *Immunobiology* 1987; **175:** 37 (abstract).
170. Clapman PB, Lester TJ, Casper ES, *et al.* Clinical pharmacology of recombinant human tumor necrosis factor in patients with advanced cancer. *J Clin Oncol* 1987; **5:** 1942–1951.
171. Creaver PJ, Plager JE, Dupere S, Huben RP, Takita H, Mittelman A. Phase I clinical trial of recombinant human tumor necrosis factor. *Cancer Chemother Pharmacol* 1987; **20:** 137–144.
172. Blick M, Sherwin SA, Rosenblum M, Gutterman J. Phase I study of recombinant tumor necrosis factor in cancer patients. *Cancer Res* 1987; **47:** 2986–2989.
173. Herrmann F. Cytokines in cancer therapy. *J Cancer Res Clin Oncol* 1989; **115:** 101–104.
174. Whitehed RP, Fleming T, Macdonald JS, *et al.* A phase II trial of recombinant tumor necrosis factor in patients with metastatic colorectal adenocarcinoma: a southwest oncology group study. *J Biol Resp Modif* 1990; **9:** 588–591.
175. Räth U, Kaufmann M, Schmid H, *et al.* Effect of intraperitoneal recombinant human necrosis factor alpha on malignant ascites. *Eur J Cancer* 1991; **27:** 121–123.
176. Schiller JH, Storer BE, Witt PL, *et al.* Biological and clinical effects of intravenous tumor necrosis factor-α administered three times weekly. *Cancer Res* 1991; **51:** 1651–1658.
177. Aulitzky WE, Tilg H, Gastl G, *et al.* Recombinant tumor necrosis factor α administered subcutaneously or intramuscularly for treatment of advanced malignant disease: a phase I trial. *Eur J Cancer* 1991; **27:** 462–467.
178. Brown TD, Goodman P, Fleming T, Macdonald JS, Hersh EM, Braun TJ. A phase II trial of recombinant tumor necrosis factor in patients with adenocarcinoma of the pancreas: a southwest oncology study group. *J Immunother* 1991; **10:** 376–378.
179. Hersh EM, Metch BS, Muggia FM, *et al.* Phase II studies of recombinant human tumor necrosis factor alpha in patients with malignant disease: a summary of the southwest oncology group experience. *J Immunother* 1991; **10:** 426–431.
180. Skillings J, Wierzbicki R, Eisenhauer E, *et al.* A phase II study of recombinant tumor necrosis factor in renal cell carcinoma: a study of the National Cancer Institute of Canada clinical trials group. *J Immunother* 1992; **11:** 67–70.
181. Semenzato G. Tumor necrosis factor: a cytokine with multiple biological activities. *Br J Cancer* 1990; **61:** 354–361.

182. Kolitz JE, Mortelsmann R. The immunotherapy of human cancer with interleukin 2: present status and future directions. *Cancer Investigation* 1991; **9**: 529–542.
183. Negrier MS, Pourrea CN, Palmer PA, *et al.* Phase I trial of recombinant interleukin-2 followed by recombinant tumor necrosis factor in patients with metastatic cancer. *J Immunother* 1992; **11**: 93–102.
184. Smith II JW, Urba WJ, Clark JW, *et al.* Phase I evaluation of recombinant tumor necrosis factor given in combination with recombinant interferon-gamma. *J Immunother* 1991; **10**: 355–362.
185. Gatanaga T, Hwang C, Kohr W, *et al.* Purification and characterization of an inhibitor (soluble tumor necrosis factor receptor) for tumor necrosis factor and lymphotoxin obtained from the serum ultrafiltrates of human cancer patients. *Proc Natl Acad Sci USA* 1990; **87**: 8781–8784.
186. Ikeda M, Sakagami H, Nishida H, Hatta Y, Fukushma Y. Inhibition of tumor necrosis factor-stimulated human peripheral blood polymorphonuclear cell iodination by sera from various cancer patients. *Anticancer Res* 1991; **11**: 1745–1750.
187. Kist A, Ho AD, Räth U, *et al.* Decrease of natural killer activity and monokine production in peripheral blood of patients treated with recombinant tumor necrosis factor. *Blood* 1988; **72**: 344–348.
188. Bartholeyns J, Lopez M, Andreesen R. Adoptive immunotherapy of solid tumors with activated macrophages: experimental and clinical results. *Anticancer Res* 1991; **11**: 1201–1204.
189. Andreesen R, Scheibenbogen C, Brugger W, *et al.* Adoptive transfer of tumor cytotoxic macrophages generated *in vitro* from circulating blood monocytes: a new approach to cancer immunotherapy. *Cancer Res* 1990; **50**: 7450–7456.

ACKNOWLEDGEMENTS. The work of the author was performed at the Department of Pharmacology, Erasmus University Rotterdam. Studies by the author were supported by the Dutch Cancer Foundation (Koningin Wilhelmina Fonds), Erasmus University Foundation Rotterdam ('Stichting Universiteitsfonds Rotterdam'), by a research fund raised by 'Supporters of the Joint Israel–Dutch Medical Research' under the auspices of the Israeli Cancer Association Tel-Aviv, Tel-Aviv, Israel and by the Emil Starkenstein Foundation, Rotterdam. The author's stay in Rotterdam was supported by the Dutch Cancer Foundation, the 'Supporters of the Joint Israel–Dutch Medical Research' and by the Erasmus University Foundation. The author is a fellow of the Lautenberg Center for General and Tumor Immunology, Hadassah Medical School, The Hebrew University Jerusalem, Israel. The author is greatly indebted to Prof. I. L. Bonta, Department of Pharmacology, Erasmus University, Rotterdam, for his continuous advice, most helpful criticism and enthusiastic support. I am indebted to Mr C. Tak for making the drawings. The typescript was prepared by Mrs B. H. M. Busscher-Lauw.

**Received 8 June 1992;**
**accepted 27 June 1992**
